# Application of Hyperspectral Imaging and Multi-Module Joint Hierarchical Residual Network in Seed Cotton Foreign Fiber Recognition

**DOI:** 10.3390/s24185892

**Published:** 2024-09-11

**Authors:** Yunlong Zhang, Laigang Zhang, Zhijun Guo, Ran Zhang

**Affiliations:** 1 School of Mechanical and Automotive Engineering, Liaocheng University, Liaocheng 252000, China; 2220230122@stu.lcu.edu.cn (Y.Z.); 2220230117@stu.lcu.edu.cn (R.Z.); 2 Institute of Information Science and Technology, Hunan Normal University, Changsha 410081, China; guozhijun@hunnu.edu.cn

**Keywords:** hyperspectral image, seed cotton foreign fiber, image recognition, preprocessing, deep learning

## Abstract

Due to the difficulty in distinguishing transparent and white foreign fibers from seed cotton in RGB images and in order to improve the recognition ability of deep learning (DL) algorithms for white, transparent, and multi-class mixed foreign fibers with different sizes in seed cotton, this paper proposes a method of combining hyperspectral imaging technology with a multi-module joint hierarchical residue network (MJHResNet). Firstly, a series of preprocessing methods are performed on the hyperspectral image (HSI) to reduce the interference of noise. Secondly, a double-hierarchical residual (DHR) structure is designed, which can not only obtain multi-scale information, but also avoid gradient vanishing to some extent. After that, a squeeze-and-excitation network (SENet) is integrated to reduce redundant information, improve the expression of model features, and improve the accuracy of foreign fiber identification in seed cotton. Finally, by analyzing the experimental results with advanced classifiers, this method has significant advantages. The average accuracy is 98.71% and the overall accuracy is 99.28%. This method has great potential for application in the field of foreign fiber identification in seed cotton.

## 1. Introduction

As one of the best textile raw materials, cotton plays an important role in the textile industry and in the lives of people [1,2]. In recent years, due to the shrinking of cotton planting land in central and eastern China, the area of cotton planting in China has been gradually reduced, from 57 million mu in 2015 to 45 million mu in 2022, a decrease of about 21%. Ranging from 5.9 million tons in 2015 to 5.97 million tons in 2022, the annual output also fluctuated in this interval, albeit maintaining a relatively stable state. The stability of production benefits from the progress of science and technology and advanced management policies [3]. However, up to now, the yield of high-quality cotton in China has still been at a low level, which directly affects economic benefits and influence in the international market [4]. The production of high-quality cotton is a major problem in China’s cotton industry. The climate in Xinjiang is dry, the temperature difference between day and night is large, and water evaporation is fast. It is generally planted with colorless transparent plastic film and black plastic film. On the one hand, it can accumulate the temperature of soil and cultivated land, while on the other hand, it can effectively reduce the evaporation of surface water and improve the survival rate of cotton [5,6]. However, colorless transparent film and black film is mixed into seed cotton in the picking stage, which becomes a key factor affecting the quality of cotton. In the process of collecting bags and processing, white label paper scraps, various color plastic strapping ropes, and transparent foam boards may be mixed into them, which also becomes a factor affecting the quality of cotton. Because the impurities are mostly transparent and white, it is difficult to distinguish them from seed cotton by the naked eye, and naked eye recognition of many kinds of impurities is very time-consuming and laborious. Therefore, a method is needed to identify colorless transparent films, plastic strapping ropes, white paper scraps, foam boards and black films in seed cotton well and conveniently, so as to improve the quality of seed cotton [7].

At present, color image recognition technology has achieved good results in the field of target recognition and classification [8,9,10,11]. However, when recognizing targets with similar colors, the recognition results are unsatisfactory. This is mainly because the color information of the captured images is close, and the deep learning (DL) training model cannot learn features and distinguish well based on the input color information. Hyperspectral imaging technology can solve the problems faced by color image recognition technology. By dividing the spectral wavelength into many bands, according to the different reflectance characteristics of each material in the same or similar bands, the hyperspectral imaging system measures each continuous band to obtain the spectral information of the object in each band, so that different materials can be distinguished [12,13,14,15].

Hyperspectral images often have rich bands, and there is information redundancy between a large number of bands, which not only increases hardware pressure but also suppresses the learning ability of the model. Numerous studies have shown that dimensionality reduction in hyperspectral images can effectively improve accuracy. Zhang et al. [16] proposed a novel unsupervised BS method based on a band correlation analysis (BCA), which can accurately identify bands with a high information content and low redundancy through iteration and correlation calculation, effectively improving the recognition rate of hyperspectral images. Ou et al. [17] proposed a band selection method based on a multi-objective cuckoo search algorithm (MOCS), which improves the focus on overall features, balances the search ability between global exploration and local exploitation, and enhances the interaction ability between individuals. It has been verified that this method has better robustness. Kang et al. [18] proposed a dimensionality reduction method combining principal component analysis and edge-preserving features, which can highlight pixel separability. Experimental verification shows that this method greatly improves the accuracy of the classifier.

With the advancement of image classification and recognition technology [19,20,21,22], a large number of scholars from different fields are gradually combining hyperspectral imaging technology with DL models to extract more feature information on the measured object, effectively improving the recognition rate of targets that are difficult to distinguish with the naked eye. Ni et al. [23] proposed an online detection method for identifying cotton mulch in hyperspectral images (HSIs) by combining a variable weighted stacked autoencoder (VW-SAE), artificial neural network (ANN), and extreme learning machine. Firstly, VW-SAE was used to extract the HSI features, then ANN was used to optimize the VW-SAE parameters, and finally, an extreme learning machine was used for classification. The overall recognition accuracy of this method was 95.58%, and after morphological processing, it can achieve accurate recognition of mulch. However, this method has lower recognition of mulch in the background and requires more computing resources, requiring high equipment requirements. Du et al. [24] proposed a method combining near-infrared spectroscopy and a convolutional neural network (CNN) and temporal convolutional network to identify foreign fibers in cotton. The original data were pre-smoothed and a feature wavelength extraction was performed, with a recognition accuracy of up to 99%. This is a promising classification algorithm. Xue et al. [25] proposed a dual-branch hierarchical residual network (Hierarchical ResNet) with an attention mechanism by improving the Hierarchical ResNet, which separates spectral and spatial dimensions for calculation purposes. This method has shown significant advantages in remote sensing land feature recognition. Collins et al. [26] used hyperspectral imaging technology combined with a three-dimensional convolutional neural network (3D-CNN) to automatically identify colon cancer and esophageal gastric cancer. The recognition accuracy of this study is as high as 93%, which provides a good technical examination method for the discovery and treatment of early cancer. Huang et al. [27] proposed a 1D CNN combined with HSIs to identify and classify textile fibers. The recognition accuracy of this study is as high as 98.6%, which provides a good technical means for archaeology, public security, and other industries. Liu et al. [28] used a 2D-CNN to identify and separate the plastic film in seed cotton, achieving an accuracy of 98.5%. Hong et al. [29] proposed a SpectralFormer model specifically designed for remote sensing, which has strong global information capture capabilities. By designing CAF and GSE blocks, the extraction of local information is improved, but the acquisition of local information is still inferior to CNN. Zhu et al. [30] designed a degradation indicator species CNN combined with HSIs to identify grassland grass species. The overall recognition accuracy of grassland species reached 98.78%, which provided a technical reference for grassland degradation monitoring and small target classification and recognition. However, there are also some defects in this study; for example, the large amount of calculations, low efficiency, excess of limited conditions in the experimental environment, and the generalization and robustness of the model need to be considered.

With the advancement of DL technology and the shortcomings of traditional classifier algorithms such as high data dependence, weak multi-classification recognition for large datasets, and poor robustness, more and more DL algorithms are gradually replacing traditional classifiers in the hyperspectral field, bringing recognition accuracy to a new level. In recent years, DL models have gradually developed towards hierarchical structures, achieving multi-layer feature extraction and effectively improving efficiency and accuracy [31,32].

Due to the weak robustness of the support vector machine (SVM) [33], ordinary CNNs lack the ability to extract multi-scale information. In order to solve the poor recognition of transparent and white impurities in cotton by color image recognition, as well as improve the recognition ability of multi-class mixed foreign fibers with different sizes in seed cotton, this paper proposes a seed cotton foreign fiber recognition method combining hyperspectral imaging technology and a multi-module joint hierarchical residual network (MJHResNet) DL algorithm. Compared with the Hierarchical ResNet structure [25], this structure has the following innovations:

(1) The channel number bisection processing of the input information of the Hierarchical ResNet is cancelled to avoid the negative impact of data randomness on the recognition results.

(2) The hierarchical structure has more parallel branches. The aim is to obtain and fuse richer scale information.

(3) Constructing a double-hierarchical residual (DHR) structure block intended to obtain and integrate richer scale information while solving the problem of gradient vanishing in layered residual structures with too many branches, resulting in the inability to obtain the maximum receptive field.

(4) The DHR module integrates a squeeze-and-excitation network (SENet) attention mechanism with the aim of reducing redundant information transmission and adaptively enhancing the model expression ability.

The method is compared with SVM, ResNet-18, 2D-CNN, Fast3D-CNN, SpectralFormer, and Hierarchical ResNet algorithms. It is concluded that the method proposed in this paper has certain advantages in terms of recognition accuracy. This method has the ability to extract multi-scale information, which can extract both global and local information and has certain application potentials in cotton processing factories, the textile industry, and other fields, which can effectively improve the quality of seed cotton, economic benefits, and influence in the cotton market. Based on its structural characteristics and the ability to extract information from small and large targets, it can also be applied in fields such as remote sensing and medicine.

## 2. Materials and Methods

### 2.1. Experimental Materials and Equipment

In this experiment, 5 kg of high-quality Xinjiang cotton, transparent films of different shapes and sizes, white paper scraps, multicolored plastic strapping ropes, white foam boards, black films, and other impurities were selected, and one or more impurities were randomly placed on the seed cotton. For the experiment, we used a hyperspectral camera (GaiaField Pro-V10E, Dualix Spectral Imaging Technology Ltd., Wuxi, Jiangsu, China) with a wavelength range of 400–1000 nm, a total of 328 bands, an exposure time of 30 ms, a frame rate of 14 frames per second, and a resolution of 2.8 nm. The experimental shooting environment was in the laboratory dark box, which can better reduce the interference of external light to improve the imaging quality. Four 50 W halogen lamps and a platform tray (the tray was 500 mm away from the hyperspectral camera objective) were used in the dark box. The experimental computer used a 12×Intel^®^ Xeon^®^ Gold 5318Y processor, 350 G hard drive, 86 G memory, NVIDIA A40 graphics card, 48 G graphics memory, python3.9, pytorch2.1.1 DL architecture, and win11 operating system.

### 2.2. Experiment Workflow

**Figure 1:**
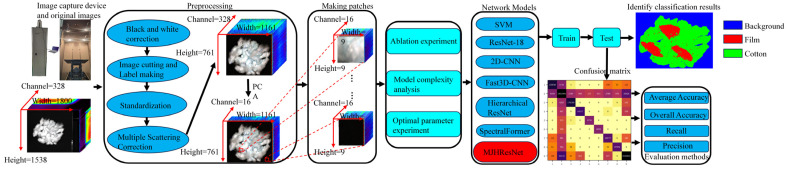
Experimental Flow Diagram.

This study was based on hyperspectral imaging and a multi-module joint hierarchical residual network to conduct research on the recognition algorithm of seed cotton foreign fibers. For the experiment, the process shown in Figure 1 was adopted. First, the collected images of seed cotton and transparent films, white paper scraps, black films, multicolored plastic strapping ropes, white foam boards, and other mixed images were preprocessed, including black-and-white correction, image cutting and label making, standardization processing, multiple scattering correction (MSC) processing, and principal component analysis (PCA) processing in sequence. After that, the preprocessed image was sliced to form a large number of small-size images of the same size and a data set was constructed for DL model training. The models included SVM, ResNet-18, 2D-CNN, Fast3D-CNN, Hierarchical ResNet, SpectralFormer, and MJHResNet, and then the average accuracy (AA), overall accuracy (OA), and recall and precision were evaluated to compare the performance of the models. Finally, in order to find the best parameters of the MJHResNet model proposed in this paper, 18 sets of experiments were carried out, and the best parameter values were obtained by comparing the AA and OA of the experimental results.

### 2.3. Preprocessing

Preprocessing is an important part of improving the accuracy of image classification and recognition. This process can remove most of the interference information, improve the quality of HSIs, and help simplify the complexity of spectral feature learning for subsequent models [34,35,36]. The preprocessing process conducted in this study was as follows.

Step 1: Black-and-white correction.

In the absence of light, the image sensor of the hyperspectral camera will also produce a small amount of electrical signal, mainly because the photodiode will release the charge, and the photodiode of each pixel acts as a capacitor at the same time, which will slowly discharge and form a so-called dark current. The dark current has a considerable influence on the imaging quality of the hyperspectral camera. The dark current will bring a lot of noise to the image, especially in a low-light environment and long-exposure environment, which greatly reduces the quality of the image. We used Formula (1) for the image black-and-white correction.

(1) $$R=\frac{L-B}{W-B}$$

where L represents the original image, B represents the image taken against a pure black background (covered by the camera cover), W represents the standard whiteboard image taken, and R represents the image after the black-and-white correction.

Step 2: Image cutting and label making.

The black-and-white corrected image was cut in three dimensions, which retained important and effective information, reduced the image size, and provided help for subsequent training. After that, ENVI5.3 was used to establish the region of interest for different categories of cut images, a spectral analysis was performed to select the band with the largest difference in reflectivity between the background, various impurities, and seed cotton, and label production was performed under this band. In these bands, various impurities had a good discrimination between them, whether in the gray single channel or the RGB pseudo color three channels, which mean that the labels produced in this band were more accurate.

Step 3: Standardization

Due to significant differences in some spectral data, it was not conducive to subsequent analysis. Standardization processing reduces the fluctuation of spectral data, which helps improve the convergence speed and learning ability of subsequent models, thereby enhancing the accuracy of subsequent processing and analysis. Using Formula (2) for standardized processing, this presents a normal distribution of spectral information with a mean of 0 and a standard deviation of 1.

(2) $$Z=\frac{A-\mu }{\sigma }$$

where Z represents the spectral data after standardized processing, A represents the input spectral data, μ represents the mean value of the input spectral data, and σ represents the standard deviation of the input spectral data.

Step 4: Multiple scattering correction

Multiple scattering correction is a data processing method mainly used to eliminate the spectral baseline shift and baseline offset caused by scattering effects. The goal of this method is to calibrate the measured spectrum to a reference spectrum, thereby reducing these irrelevant changes and improving the accuracy and reliability of the analysis. Firstly, we used Formula (3) to calculate the average spectrum of the image, then we used Formula (4) to perform a univariate linear regression calculation on the average spectrum and to calculate the baseline shift (regression constant) and baseline offset (regression coefficient) of each spectrum relative to the average spectrum, and finally we used Formula (5) to complete the dispersion correction [34].

(3) $$\bar{Z}=\frac{\sum_{i=1}^{n}Z_{i,j}}{n}$$

(4) $$Z_{i}=m_{i}\bar{Z}+b_{i}$$

(5) $$Z_{i\;(MSC)}=\frac{Z_{i}-b_{i}}{m_{i}}$$

where $Z_{i,j}$ represents the spatial position of the hyperspectral image sample of seed cotton foreign fiber, n represents the spectral band, $\bar{Z}$ represents the average spectral vector obtained by averaging the original spectral data of all samples at each wavelength, $Z_{i}$ is a 1×n dimensional matrix, which represents the spectral vector of a single sample, $b_{i}$ and $m_{i}$ represent the baseline translation and offset of each sample obtained by the unary linear regression of the near-infrared spectrum $Z_{i}$ and the average spectrum $\bar{Z}$, respectively, and $Z_{i\;(MSC)}$ represents the hyperspectral image that has completed the multiple scattering correction.

Step 5: Principal component analysis.

The information between each band in hyperspectral images has a strong correlation, which can cause a large amount of redundant information, increase the storage and computing pressure of hardware devices, and increase the risk of overfitting. Dimensionality reduction techniques can solve the catastrophic problem of redundant and complex high-dimensional data. PCA and autoencoders are both technical methods for data dimensionality reduction. Compared with autoencoders, PCA has the advantages of fast calculation speed, low model complexity, and strong interpretability. It maps high-dimensional spatial features to low-dimensional space through linear transformation, which preserves as much original data as possible while reducing image dimensionality and noise, resulting in image compression to a large extent. The dimensionality reduction of hyperspectral data in this article is beneficial for subsequent data analysis and visualization processing. We centralized the input sample set $D=\left(z^{\;(1)},z^{\;(2)},\ldots ,z^{\;(m)}\right)$ using PCA, then calculated the covariance matrix $ZZ^{T}$ of the sample and performed eigenvalue decomposition. The eigenvector $\left(w_{1},w_{2},\ldots ,\right.\left.w_{p}\right)$ corresponding to the largest p eigenvalues was taken out, and all the eigenvectors were standardized to form the eigenvector matrix W, and then each sample $z^{\;(i)}$ was converted into a new sample $x^{\;(i)}$, and finally the output sample set $X=\left(x^{\;(1)},x^{\;(2)},\ldots ,x^{\;(m)}\right)$ was obtained (Formulas (6) and (7)). This paper specifies a principal component proportion threshold b. Then p can be obtained by Formula (8).

(6) $$z^{\;(i)}=z^{\;(i)}-\frac{1}{m}\sum_{j=1}^{m}z^{\;(j)},\;(i,\;j=1,2,\ldots ,m)$$

(7) $$x^{\;(i)}=W^{T}z^{\;(i)}$$

(8) $$\frac{\sum_{i=1}^{p}\lambda_{i}}{\sum_{i=1}^{n}\lambda_{i}}≧b$$

where $z^{\;(i)}$, $z^{\;(j)}$ are n dimensional samples of the image to be processed, $x^{\;(i)}$ is p dimensional samples, and $W^{T}$ represents the transpose of the eigenvector matrix W.

### 2.4. Multi-Module Joint Hierarchical Residual Network

**Figure 2:**
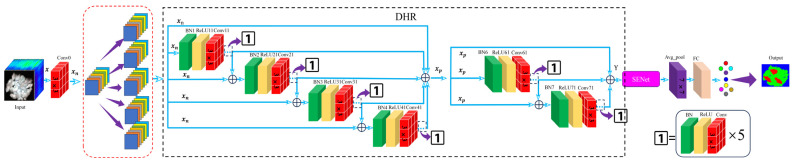
MJHResNet structure diagram.

At present, many CNNs mainly improve the information extraction ability of their models by increasing the number of network layers. As the number of network layers increases, there will inevitably be gradient disappearance and network degradation, which will lead to problems such as increased training difficulty and a sudden decrease in accuracy, thus hindering training. Additionally, due to the high-dimensional nature of HSIs, which contain a large amount of data information, extracting information becomes more cumbersome, making the degradation of network performance more apparent [25,31]. However, the identity mapping structure of residual structures [37] and the multi-branch structure of hierarchical structures [32] can effectively solve the problems of gradient disappearance and network degradation and single-scale information. Given the good performance of the Hierarchical ResNet composed of a residual structure and hierarchical structure in image recognition and classification, and in order to better extract multi-scale information from HSIs and solve the problem of identifying seed cotton foreign fibers, this paper proposes an innovative MJHResNet model. The model is shown in Figure 2. It contains a Conv0 block, a DHR structure, a SENet attention structure, a 7 × 7 average pooling layer, and a fully connected layer.

#### 2.4.1. Double-Hierarchical Residual Structure

This MJHResNet designs a novel DHR structure, which concatenates two hierarchical residual structures of different sizes and adds branches of the hierarchical residual structure to improve the model’s ability to extract multi-scale features. However, research has found that increasing the number of branches in the hierarchical residual structure without restraint does not necessarily result in rich scale information, but rather increases the risk of gradient disappearance. So, after adding 5 branches to this structure, the other two branches are combined to form a second hierarchical residual structure, which is connected to the first hierarchical residual structure; each branch that performs convolution operations contains 6 BN-ReLU-Conv convolution blocks, as shown in the black dashed box in Figure 2. This structural design will reduce the risk of gradient disappearance, obtain richer scale information, and obtain feature maps with a larger receptive field. At the same time, the input information of each branch of the structure is no longer subjected to channel number split processing, which can easily generate data randomness. The key information may not be learned by the deep network, so full channel information is adopted, as shown in the red dashed box in Figure 2. The input information x is Conv0 to obtain $x_{n}$, which is then passed into the DHR structure. After convolution operations C(·) and feature information cascading ⊕, a multi-scale feature map is obtained Y. The information transformation is shown in the Formulas (9)–(13), and the detailed parameter information is shown in Table 1.

The relationship is as follows:

(9) $$y_{i}=\left\{\begin{array}{c} x_{n}\\ {C\;(x}_{n})\\ \begin{array}{c} C\left(x_{n}+y_{1}\right)\\ C\;(x_{n}+y_{2})\\ C\;(x_{n}+y_{3}) \end{array} \end{array}\;(i=0,1,2,3,4)\right.$$

(10) $$x_{p}=\sum_{i=0}^{4}y_{i}$$

(11) $$y_{j}=\left\{\begin{array}{c} x_{p}\\ C\;(x_{p})\\ C\;(x_{p}+y_{6}) \end{array}\;(j=5,6,7)\right.$$

(12) $$Y=\sum_{i=5}^{7}y_{j}$$

where $y_{i}$ is the output information of each branch in the first hierarchical residual structure, and $y_{j}$ is the output information for each branch in the second hierarchical residual structure. $x_{p}$ represents the fusion information of the first hierarchical residual structure.

#### 2.4.2. Squeeze-and-Excitation Network Attention

**Figure 3:**
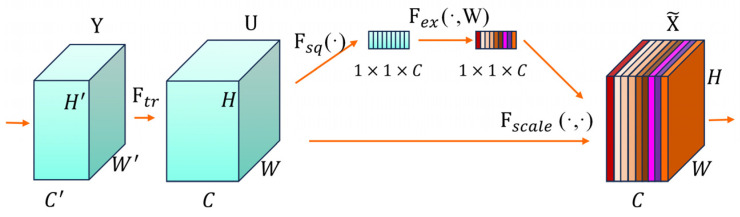
SENet structure diagram.

Due to the characteristics of hierarchical residual structures, the fused multi-scale feature maps contain a large amount of redundant information, which can affect the learning ability of the model [38]. SENet [39] can adaptively learn the importance of different feature channels, reducing the weight of invalid or unimportant feature maps, thereby reducing information redundancy and enhancing the model’s expression ability. The principle is to sequentially use $F_{tr}$ conversion, Squeeze, Excitation, and Fscale, as shown in Figure 3.

$F_{tr}$ conversion converts input Y to output U through a convolution operation, as shown in Formula (13).

(13) $$U_{C}=V_{C}\times Y$$

where $V_{C}$ represents the C-th convolutional kernel, $U_{C}$ represents the C-th input of U, and C represents the number of channels.

Squeeze is a global average pooling process that converts the input of H × W × C into an output of 1 × 1 × C, obtaining the global information of the input information U, as shown in Formula (14) [39].

(14) $$z_{C}=F_{sq}\;(U_{C})=\frac{1}{W\times H}\sum_{i=1}^{W}\sum_{j=1}^{H}U_{C}\;(i,j)$$

where W represents the width of feature map U, H represents height, and $U_{C}\;(i,j)$ represents the point in $U_{C}$ where the width is i, height is j, and $z_{C}$ represents the output 1 × 1 × C.

Excitation is a two-time full connection process. The first full connection is used for dimensionality reduction, and then the nonlinear transformation is performed by the ReLU activation function. The second full connection is restored to the original dimension, and is mapped to 0 and 1 by the sigmoid activation function to obtain the weight of each channel [36], as shown in Formula (15).

(15) $$s=F_{ex}\left(z,W\right)=\sigma \left(g\left(z,W\right)\right)=\sigma (W_{2}\delta \left(W_{1}z\right))$$

where $W_{1}$ represents the dimension reduction parameter, z represents the sum of $z_{C}$, $W_{2}$ represents the dimension-up parameter. δ represents ReLU, σ represents sigmoid, and s represents the weight of the output; the size is 1 × 1 × C.

The Fscale operation multiplies the obtained weight s with the original feature map U to complete the recalibration of the original features on the channel dimension [39], as shown in Formula (16).

(16) $$\tilde{X_{C}}=F_{scale}\left(U_{C},s_{C}\right)=s_{C}\cdot U_{C}$$

where $s_{C}$ represents the weight, $\tilde{X_{C}}$ represents the final output, and the size is H × W × C.

The SENet module is fused with the DHR module to form the DHR–SE module. This module can not only improve the extraction ability of multi-scale information of the network, but also avoid the disappearance of gradient, reduce information redundancy, and improve the recognition rate of foreign fibers in seed cotton.

### 2.5. Model Evaluation Methods

To compare the performance of network models, this paper evaluates them using methods such as Recall, Precision, OA, and AA. Recall and precision reflects the model’s ability to recognize positive and negative samples (Formulas (20) and (21)). The AA method reflects the average level of prediction by the model for each category, which is the sum of the accuracies of all the categories divided by the number of categories (Formula (18)). The OA method reflects the overall prediction level of the model, which is determined by the proportion of correctly identified samples of all the categories to the total samples of all the categories (Formula (19)). All four perspectives presented are the most important methods for evaluating the quality of the model currently.

These evaluation methods are as follows:

(17) $$Recall=\frac{TP}{TP+FP}$$

(18) $$Precision=\frac{TP}{TP+FN}$$

(19) $$AA=\frac{\sum_{i=1}^{C}Recall}{C}\;(i=1,2,\ldots ,C)$$

(20) $$OA=\frac{\sum_{i=1}^{C}TP}{\sum_{i=1}^{C}\;(TP+FP)}=\frac{\sum_{i=1}^{C}TP}{\sum_{i=1}^{C}\;(TP+FN)}\;(i=1,2,\ldots ,C)\;$$

where i represents category, C represents the total number of categories, TP represents the number of samples that are actually positive examples and are judged as positive examples by the classifier, FP represents the number of samples that are actually negative and judged as positive by the classifier, and FN represents the number of samples that are actually positive but judged as negative by the classifier.

## 3. Results and Discussion

A total of 50 HSIs of seed cotton mixed with various impurities were taken in the dark box environment. The image specification was 1800 × 1538 × 328.

### 3.1. Preprocessing

After preprocessing, image samples can effectively improve spectral features, reduce interference information, enhance spectral information, and improve the analytical expression ability of subsequent models.

#### 3.1.1. Image Cutting and Label Making

**Figure 4:**
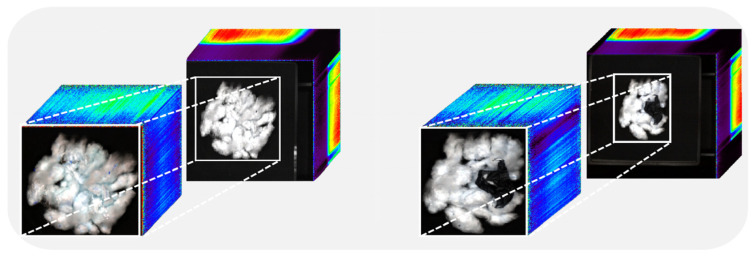
Hyperspectral image cropping image.

The black-and-white corrected image with a size of 1800 × 1538 × 328 was subjected to redundant black background cutting. The processing procedure is shown in Figure 4. After cutting, images with different spatial sizes and the same spectral size, such as 971 × 841 × 328, 1161 × 761 × 328, and 1241 × 1081 × 328, were obtained, and the cut images were labeled. After cutting, a size of 971 × 841 × 328, 1161 × 761 × 328, 1241 × 1081 × 328 and other images with different spatial dimensions and the same spectral dimension as in Figure 4 were achieved, and the cut images were labeled. There were nine label categories: seed cotton, plastic film, paper scraps, background, black film, transparent plastic rope, white plastic rope, red plastic rope, and foam board.

**Figure 5:**
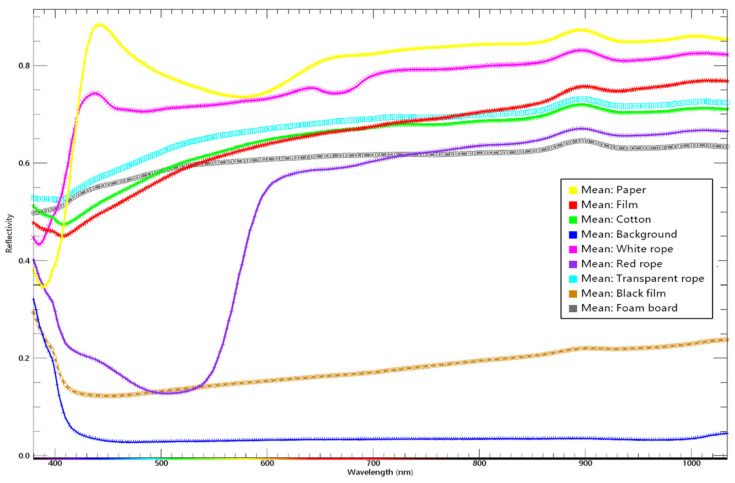
Spectral curve diagram.

Regions of interest were established and spectral curves generated for each category. As shown in Figure 5, the wavelength range with the greatest difference in reflectance between various impurities and seed cotton was identified, and labels were created at wavelengths with significant differences.

#### 3.1.2. Standardized Processing

**Figure 6:**
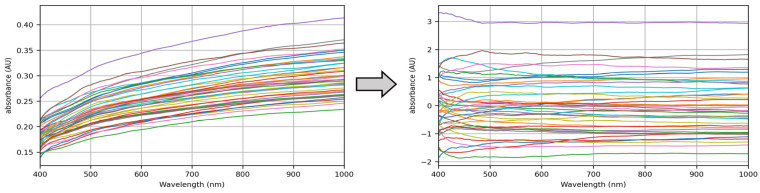
Visualization of standardized processing.

After standardization processing, the image data had a regular distribution (Figure 6), symmetrically distributed around 0.

#### 3.1.3. MSC Processing

**Figure 7:**
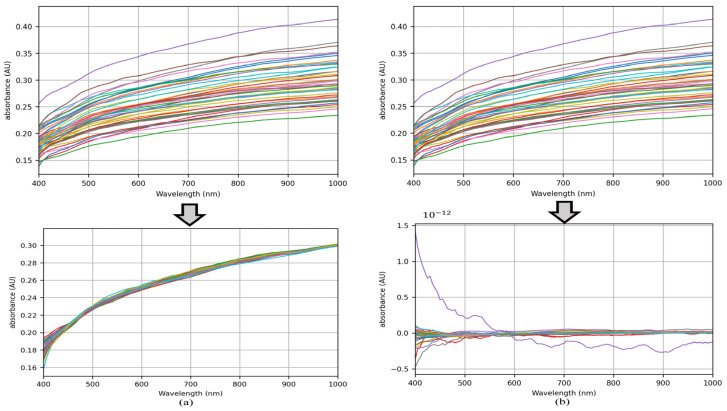
Visualization of MSC processing. (**a**) Visualization image after MSC processing only; (**b**) Visualization image after standardization and MSC processing in sequence.

Figure 7 shows the visualization of the processing for MSC. The vertical absorbance is obtained by logarithmic conversion formula $-\log_{10}\;(\frac{I}{I0})$ between the measured light intensity I (MSC corrected light intensity) and the reference light intensity I0 (light intensity without absorption). It can be seen from Figure 7a that the sample data are more concentrated after MSC, which reduces the sample difference. It can be seen from Figure 7b that most of the data are concentrated near 0 after standardization and MSC. The data are simpler and more regular, which effectively eliminates the influence of scattering level difference and is conducive to the analysis and processing of sample data by later models.

#### 3.1.4. PCA Processing

**Figure 8:**
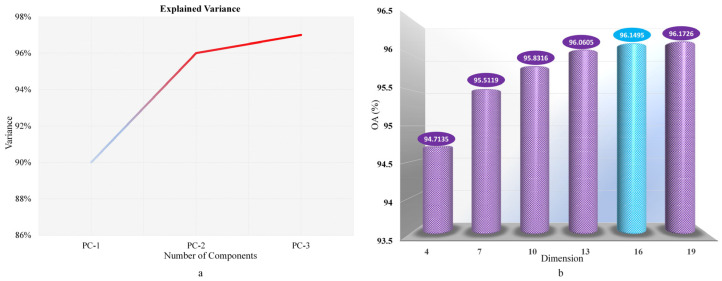
Experimental diagram of PCA. (**a**) Explanation of variance of the first three principal components; (**b**) OA diagram of PCA experiment.

According to the analysis in Figure 8a, the variance explanatory values of the first three principal components are 97%, indicating that the first three principal components can effectively capture the variability of the original data and can be used to represent the original data. The experimental dimensions retain 4, 7, 10, 13, 16, and 19 dimensions, respectively. As shown in Figure 8b, after experimental verification, After the experimental verification shown in Figure 8b, under the same experimental conditions, the OA of each group obtained by only changing the number of retained dimensions showed that the OA varied greatly from 4 to 13 dimensions and tended to stabilize from 16 to 19 dimensions. In this study, 16 dimensions were selected as the experimental benchmark to balance accuracy and device performance, which not only retained the original information to the greatest extent but also significantly reduced the image dimension.

### 3.2. Ablation Experiment on Sample 1 and Sample 2

In this section, ablation experiments were carried out on sample 1 and sample 2. By increasing or decreasing the branches of the DHR structure and increasing the SENet, experiments were carried out one by one to evaluate the effectiveness of a double residual structure instead of a single residual structure and a fusion SENet module. Table 2 shows the results of the ablation experiments, and the training parameters of all the models are the same.

According to the experimental results shown in Table 2, exceeding a certain number of branches in the hierarchical residual structure does not increase the recognition accuracy, but has the opposite effect, mainly due to the gradient vanishing caused by the excessive number of convolutions used to obtain advanced features. Using a double-hierarchical residual structures instead of a single hierarchical residual structure with the same number of convolutional branches can effectively improve accuracy. The integrated SENet module is beneficial for improving recognition accuracy and enhancing the feature expression ability of the model.

### 3.3. Model Complexity Analysis

This section conducted a complexity analysis of deep learning models, using two metrics: parameters and FLOPs. The specific data are shown in Table 3.

According to the data shown in Table 3, MJHResNet has advantages in terms of parameter count and FLOPs compared to ResNet-18, 2D-CNN, and Fast3D CNN. However, compared to HResNet, MJHResNet has higher complexity, mainly due to having more convolutional branches and modules to obtain multi-scale information.

### 3.4. Optimal Parameter Experiment

**Figure 9:**
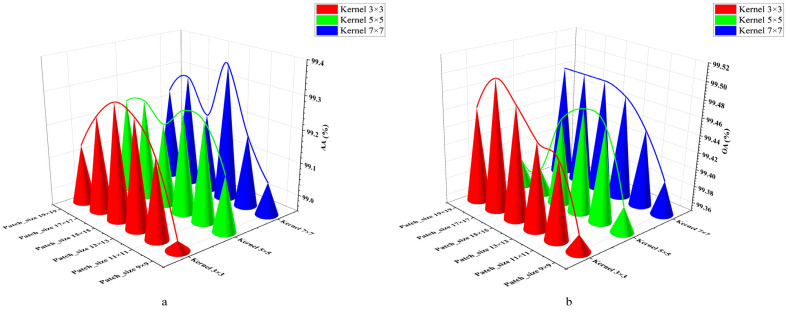
Experimental diagram of optimal parameter distribution. (**a**) AA values corresponding to different sizes of convolution kernels and patches; (**b**) OA values corresponding to different sizes of convolution kernels and patches.

**Figure 10:**
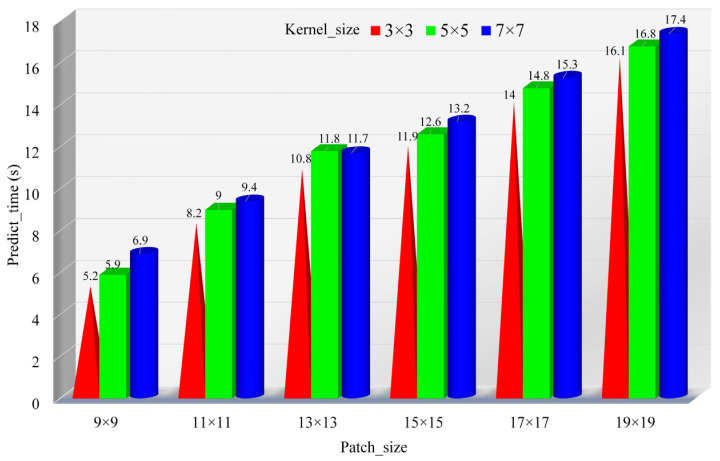
Prediction time of each experimental parameter.

In order to find the optimal parameters for MJHResNet, this study conducted 18 experiments on sample (4) by changing the size of the convolution kernels (3 × 3, 5 × 5, 7 × 7) and patch sizes (9 × 9, 11 × 11, 13 × 13, 15 × 15, 17 × 17, 19 × 19). The experimental results shown in Figure 9 and Figure 10 show that the highest AA value (99.36%) is achieved at the convolution kernel of 7 × 7 and the patch size of 13 × 13, with a prediction time of 11.7 s. The highest OA value (99.50%) is achieved at the convolution kernel of 3 × 3 and the patch size of 17 × 17, with a prediction time of 14 s. The optimal value is evaluated comprehensively at the convolution kernel of 7 × 7 and the patch size of 13 × 13 (AA: 99.36%; OA: 99.47%). Large convolutional kernels and patch sizes not only improve accuracy, but also increase the running pressure on computers, resulting in a longer computation time and affecting efficiency. The convolution kernel is 3 × 3, the patch size is 9 × 9, and the prediction time is 5.2 s. Finally, considering the performance bottleneck of the computer in this study, we chose to sacrifice a small part of accuracy to improve the computational efficiency without affecting the target extraction. In this paper, we chose a 3 × 3 convolution kernel and a 9 × 9 patch for the experiments. Afterwards, six experiments were conducted with learning rates of 1.0, 0.1, 0.01 (1.0, 0.1), (1.0, 0.01), and (0.1, 0.01). According to the experimental results shown in Table 4, the combination of learning rates (1.0, 0.1) yielded the best results.

### 3.5. Model Training

In this experiment, 50 preprocessed image samples (including transparent film, paper scraps, black film, transparent plastic strapping rope, white plastic strapping rope, red plastic strapping rope, white foam board, cotton, and background) were sliced to form a large number of 9 × 9 × 16 patches, and a data set was constructed. We used 8% of the dataset samples as the training set, which was imported into SVM, ResNet-18, 2D-CNN, Fast3D-CNN, Hierarchical ResNet, SpectralFormer, and MJHResNet models for recognition and classification experiments.

#### 3.5.1. Model Parameter Settings

This model uses an Adadelta optimizer, ReLU activation function, CrossEntropyLoss function, and has been trained for 100 epochs. The first 70 epochs have a learning rate of 1.0, and the last 30 epochs have a learning rate of 0.1. The batch size is 1024.

#### 3.5.2. Model Training Results

**Figure 11:**
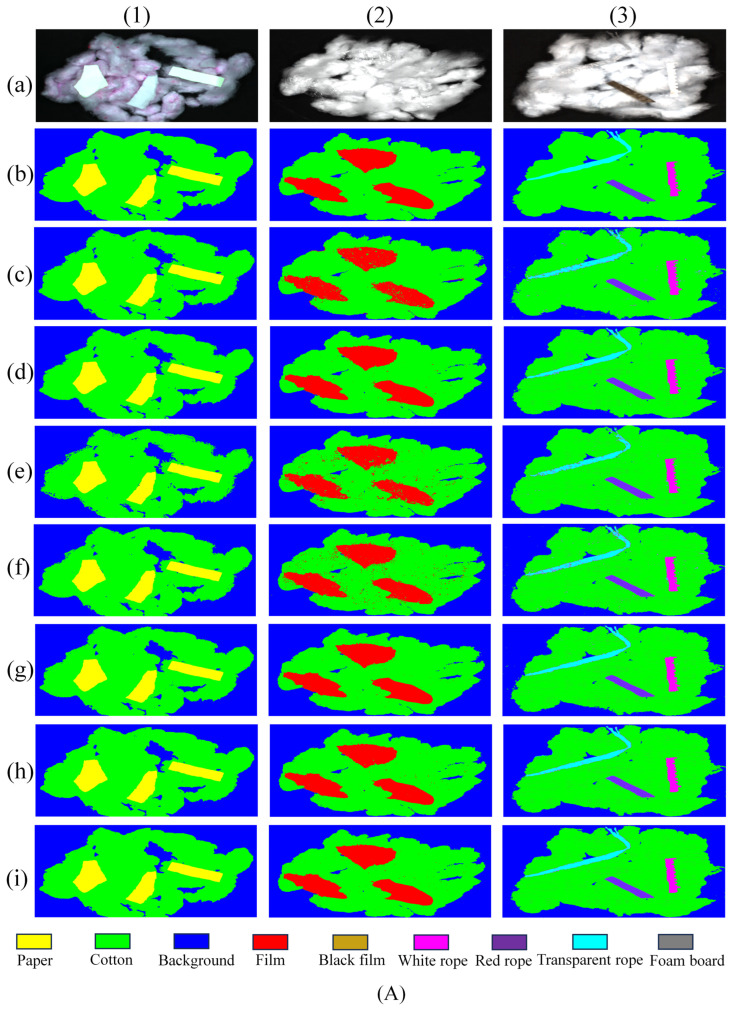

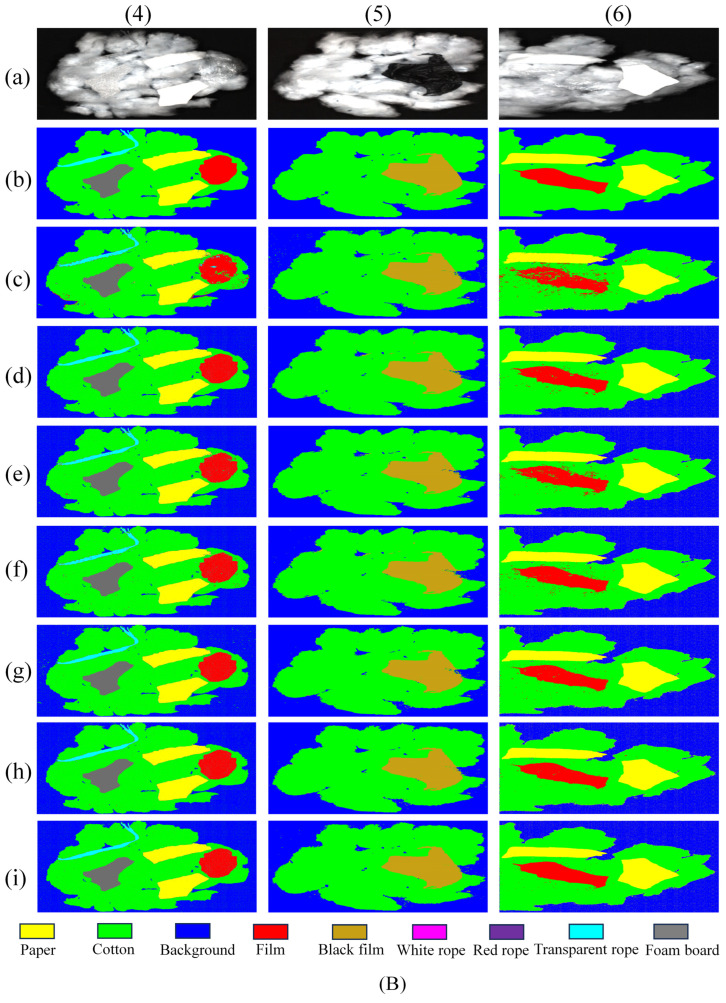
Classification diagram for identifying different fibers in seed cotton. (**A**) the original image sample (1), sample (2), sample (3) experimental results; (**B**) the original image sample (4), sample (5), sample (6) experimental results; (**a**) Original image; (**b**) Label; (**c**) SVM; (**d**) ResNet-18; (**e**) 2D-CNN; (**f**) Fast3D-CNN; (**g**) Hierarchical ResNet; (**h**) SpectralFormer; (**i**) MJHResNet.

In Figure 11, the recognition and classification results of the MJHResNet model proposed in this paper and the comparison models SVM [33], ResNet-18 [37], 2D-CNN [28], Fast3D-CNN, SpectralFormer [29], and Hierarchical ResNet [25] are shown. From the recognition result graph, it can be seen that the MJHResNet model has the best performance, with a more accurate recognition of various impurities and fewer false recognitions of impurities, proving that this method is scientifically effective. Each algorithm has good recognition performance for sample (1) in Figure 11A and sample (5) in Figure 11B. In the result graphs of the other samples, the recognition ability of Fast3D-CNN is weaker than that of other DL models, with more misrecognition areas. SVM has weak generalization and poor recognition of impurities such as plastic film and transparent rope.

**Figure 12:**
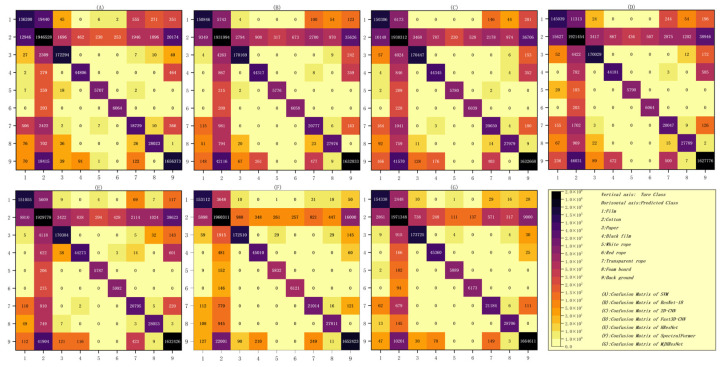
Confusion matrix of all models. (**A**) SVM; (**B**) ResNet-18; (**C**) 2D-CNN; (**D**) Fast3D-CNN; (**E**) Hierarchical ResNet; (**F**) SpectralFormer; (**G**) MJHResNet.

According to the confusion matrix of the seven types of models shown in Figure 12, it can be intuitively seen that all the foreign fibers identified by the seven types of models are mistakenly identified as seed cotton, mainly because the foreign fibers are located on the seed cotton, which is caused by the inaccurate recognition of some edges. At the same time, according to the confusion matrix, the recognition results of each type of model can be calculated statistically (Table 5). In Table 5, it can be seen that, compared with other impurities, each type of model has relatively weak recognition of transparent film and transparent rope, mainly because the light will pass through them and shine on the seed cotton, which is similar to the reflectivity change of the seed cotton, and the difference is small. According to the obtained results, it can be seen that the MJHResNet model improved various indicators compared to the Hierarchical ResNet model, and compared with the advanced SpectralFormer classifier it also has advantages, proving that our improvement is successful. SVM has weak robustness and significant differences in recognition accuracy between different categories, resulting in the lowest AA of 94.98%. In addition, compared with the other six models, the MJHResNet model proposed in this paper has better performance in the experiment of seed cotton foreign fiber recognition and classification, with the AA and OA reaching 98.71%, 99.28%, respectively. Furthermore, the recall and precision of each category are both above 96%.

**Figure 13:**
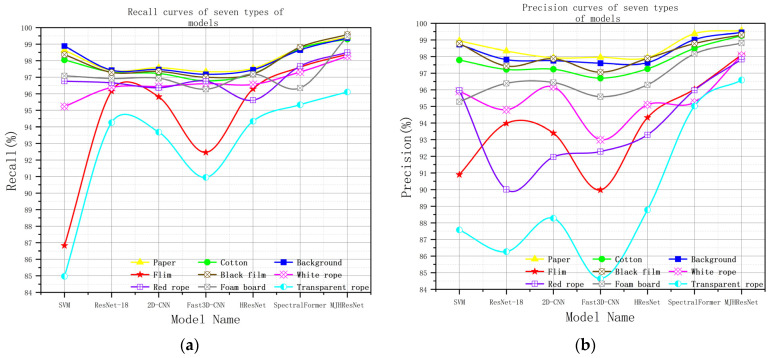
Recall and Precision Description Charts of all models. (**a**) Recall curves of seven types of models; (**b**) Precision distribution map of seven types of models.

In Figure 13, the horizontal and vertical coordinates show the recognition recall and precision of seven types of models for nine types of samples. In Figure 13a, it can be clearly seen that each type of model has relatively weak recognition of transparent rope, red rope, and white rope, especially for the SVM model, which may be affected by the number of samples. Due to the small volume of transparent rope, white rope, and red rope, the number of samples is small, which leads to insufficient network learning. In Figure 13b, it can be seen that the precision values of the first four types of models fluctuate greatly, reflecting weaker robustness of the models, while the latter three types of models are more concentrated and have better robustness. From Figure 13a,b, it can be seen that the MJHResNet model has significant advantages compared to the other six types of models, fully demonstrating the feasibility of this model.

The experimental results show that the MJHResNet model proposed in this paper is feasible. It can complete the task well in the work of seed cotton foreign fiber classification and recognition, and the pretreatment process promotes the accurate recognition and classification of seed cotton foreign fiber to a certain extent. It has great potential for application in fields such as medicine, the textile industry, and industrial materials.

## 4. Conclusions

In order to solve the problem of the difficulty of identifying white paper scraps, foam boards, colorless transparent films, and other foreign fibers in seed cotton, this paper proposes a method combining hyperspectral imaging technology in the 400–1000 nm spectral range with the MJHResNet algorithm. This method performs black-and-white correction, image cutting, standardization processing, MSC processing, and PCA processing on the original image in advance to effectively enhance the spectral feature information. This method has shown good performance in identifying seed cotton foreign fibers, with an AA and OA of 98.71% and 99.28%. It can complete the recognition and classification of seed cotton foreign fibers excellently, and has good generalization ability and robustness. It has great potential for application in the field of seed cotton foreign fiber recognition. Due to the consideration of device performance, the experiment did not use large convolutional kernels, large patch sizes, and data parallel computing. In addition, whether the accuracy could be further improved by using a spatial–spectral combination will be discussed. In future research, we will expand the dataset and use more types of seed cotton fibers to design and train an optimal autoencoder for more complex tasks. Moreover, the hardware equipment will be upgraded and a spatial–spectral combination and multi-scale convolution kernel will be used. Further research will be carried out in the direction of large patch size experiments and multi-GPU parallel computing, which will increase the accuracy, shorten the prediction time, and provide a more accurate and efficient method for identifying seed cotton foreign fiber.

## Figures and Tables

**Table 1:** MJHResNet parameters.

Layer Name	Output Shape	Convolution Kernel	Step	Padding
Input	9 × 9 × 16			
Conv0	7 × 7 × 64 (1)	3 × 3 × 16 (1)	1	(0,0)
Conv1	7 × 7 × 64 (6)	3 × 3 × 64 (6)	1	(1,1)
Conv2	7 × 7 × 64 (6)	3 × 3 × 64 (6)	1	(1,1)
Conv3	7 × 7 × 64 (6)	3 × 3 × 64 (6)	1	(1,1)
Conv4	7 × 7 × 64 (6)	3 × 3 × 64 (6)	1	(1,1)
Conv6	7 × 7 × 64 (6)	3 × 3 × 64 (6)	1	(1,1)
Conv7	7 × 7 × 64 (6)	1 × 1 × 64 (6)	1	(1,1)
SENet	7 × 7 × 128			
Avg_pool	1 × 1 × 128	7 × 7 × 128		
Output	Class (9)			

**Table 2:** Ablation experiment.

Method	Sample 1		Sample 2	
	AA	OA	AA	OA
The first hierarchical residual structure in the DHR structure	98.1653	98.1888	97.0243	97.3698
Single multi-branch residual structure replaced by DHR	97.6769	98.2116	96.7951	96.129
DHR	98.209	98.3196	97.8793	98.2809
DHR + SENet	**98.4296**	**98.43**	**98.0765**	**98.6844**

With the best results highlighted in bold.

**Table 3:** Model complexity analysis.

Model	ResNet-18	2D-CNN	3D-CNN	HResNet	MJHResNet
Parameters (M)	11.4	2.16	2.43	0.46	1.06
FLOPs (G)	561.5	41.3	40.6	22.8	33.6

**Table 4:** Optimal learning rate results.

Group	Epoch_a	Epoch_b	Learning Rate_a	Learning Rate_b	AA	OA
1	70	30	1.0	1.0	98.21%	99.03%
2	70	30	0.1	0.1	98.77%	99.21%
3	70	30	0.01	0.01	98.62%	99.12%
4	70	30	1.0	0.1	**98.98%**	**99.37%**
5	70	30	1.0	0.01	98.83%	99.21%
6	70	30	0.1	0.01	98.67%	99.28%

With the best results highlighted in bold.

**Table 5:** Recognition result data for all models.

		Film	Cotton	Paper	Black Film	White Rope	Red Rope	Transparent Rope	Foam Board	Background	AA	OA
SVM	Recall	86.8235	98.0455	98.6301	98.3645	95.2278	96.7608	84.9696	97.0863	98.8814	94.9766	97.903
	Precision	90.9006	97.7881	98.9456	98.7808	95.8998	95.9646	87.5719	95.2839	98.7247		
ResNet-18	Recall	96.1599	97.3134	97.4137	97.2909	96.3791	96.6651	94.261	96.9235	97.4283	96.6483	97.2988
	Precision	93.9774	97.2228	98.3318	97.43	94.7973	90.0015	86.2653	96.3892	97.8117		
2D-CNN	Recall	95.8156	97.2287	97.5728	97.3524	96.4459	96.3619	93.6848	96.9339	97.4663	96.5403	97.2641
	Precision	93.3931	97.237	97.9277	97.8681	96.173	91.9598	88.2743	96.4427	97.7488		
Fast3D-CNN	Recall	92.458	96.783	97.334	96.992	96.613	96.761	90.949	96.276	97.174	95.7043	96.7677
	Precision	89.9768	96.6984	97.952	97.0584	92.9971	92.2843	84.6436	95.5835	97.6		
Hierarchical ResNet	Recall	96.2931	97.2018	97.5367	97.1944	96.5627	95.6119	94.3426	97.1903	97.4518	96.5984	97.2646
	Precision	94.3262	97.2587	97.9325	97.8863	95.1027	93.2897	88.7803	96.3028	97.6254		
SpectralFormer	Recall	97.6044	98.7398	98.7538	98.8123	97.3135	97.6703	95.3362	96.3519	98.6456	97.692	98.6204
	Precision	96.0401	98.4894	99.3733	98.7755	95.2474	95.9705	95.0215	98.1611	99.0187		
MJHResNet	Recall	**98.386**	**99.296**	**99.449**	**99.581**	**98.265**	**98.5**	**96.107**	**99.453**	**99.373**	**98.7122**	**99.2835**
	Precision	**98.097**	**99.2573**	**99.5542**	**99.3038**	**98.0683**	**97.8288**	**96.5851**	**98.809**	**99.4507**		

With the best results highlighted in bold.

## Data Availability

The data contained within the article and the data used in this study are available from the corresponding author on reasonable request.
